# Implications to payers of switch from hospital-based intravenous immunoglobulin (IVIg) to home-based subcutaneous immunoglobulin (SCIg) therapy in patients with primary immunodeficiencies (PID) and secondary immunodeficiencies (SID) in Canada

**DOI:** 10.1186/1710-1492-10-S1-A42

**Published:** 2014-03-03

**Authors:** William C Gerth, Stephen D Betschel, Arthur S Zbrozek

**Affiliations:** 1 W. C. Gerth & Associates, Shrewsbury, New Jersey, 07702, USA; 2 University of Toronto, Toronto, Ontario, Canada, M5B 1W8; 3 CSL Behring, King of Prussia, Pennsylvania, 19406, USA

## Background

A nurse shortage in Canada is contributing to large amounts of paid/unpaid overtime and increased labor-related expenditures. Shifting PID/SID patients from hospital-based IVIg to less labor-intensive home-based SCIg can improve their quality of life and treatment satisfaction [1] and may improve efficient allocation of nurse staff. The objectives for this study were to estimate the return on investment in the upfront cost in nursing time for patient training and the number of PID/SID patients needed to be switched to SCIg to gain one full-time nurse equivalent (FTE).

## Methods

The return on investment was estimated by multiplying the hourly nurse compensation ($57.58 = $104,440 ÷ 1,813) for wage and benefits for a general duty nurse in British Columbia by the number of nursing hours in year 1 and subsequent years required for each route of administration. The number of patients needed to be switched to SCIg to gain one nurse FTE was estimated by dividing the number of work hours in a typical year for publicly-employed nurses (1,813 = 37 hours x 49 weeks), based on a report prepared for the Canadian Federation of Nurses Unions [2], by the average annual savings in nursing time over 3 years in Canada based on Martin et al [3]. (49.2 hours). Sensitivity analyses were performed adjusting the number of hours in a typical year and nurse compensation.

## Results

The initial investment in year 1 for nurse time to switch one patient to home-based SCIg care would be $691 (6 hours training, 6 hours monitoring) and $345 (6 hours monitoring only) annually thereafter to offset $3,294 (57.2 hours set-up and monitoring) of hospital-based nursing time annually. There would need to be 37 IVIg patients switched to home-based SCIg to gain one nurse FTE annually. The annual net saving for these 37 patients in nursing time in year 1 would be $96,297 and $109,080 annually in subsequent years. Sensitivity analysis show that as the number of hours in a work year decline, as a consequence of a shorter work week and/or fewer weeks in the year, the number of switched patients needed to gain one FTE decreases.

## Conclusions

The shift from labor-intensive hospital-based IVIg to less labor-intensive home-based SCIg therapy has the potential to reduce overall healthcare costs, alleviate nurse shortages, and improve efficient allocation of nurse staff in Canada. Health care professionals should consider advocating for home-based SCIg therapy for PID/SID patients when clinically appropriate.

## References

[B1] Jones C Rojavin M Baggish J Patients with primary immunodeficiency receiving subcutaneous immune globulin Hizentra maintain health-related quality of life and treatment satisfaction in a multicentre extension study of efficacy, tolerability and safety J Health Serv Res 2012 3 41 47

[B2] Lasota M Trends in own illness or disability-related absenteeism and overtime among publicly-employed registered nurses - summary of key finding Report prepared by Informetrica Limited for Canadian Federation of Nurses Union 2009 Ottawa

[B3] Martin A Lavoie L Goetghebeur M Schellenberg R Economic benefits of subcutaneous rapid push versus intravenous immunoglobulin infusion therapy in adult patients with primary immune deficiency Transfus Med 2013 10 55 60 10.1111/j.1365-3148.2012.01201.x 23167310 PMC3580879

